# Supplementary material for: Ecological significance of extracellular vesicles in modulating host-virus interactions during algal blooms
Source: ISME J. 2021 Jun 4;15(12):3714–21. doi: 10.1038/s41396-021-01018-5 (PMC8630046; doi:10.1038/s41396-021-01018-5)
Supplement: Supplementary file 1 — Figure S1 [file 41396_2021_1018_MOESM1_ESM.pdf]

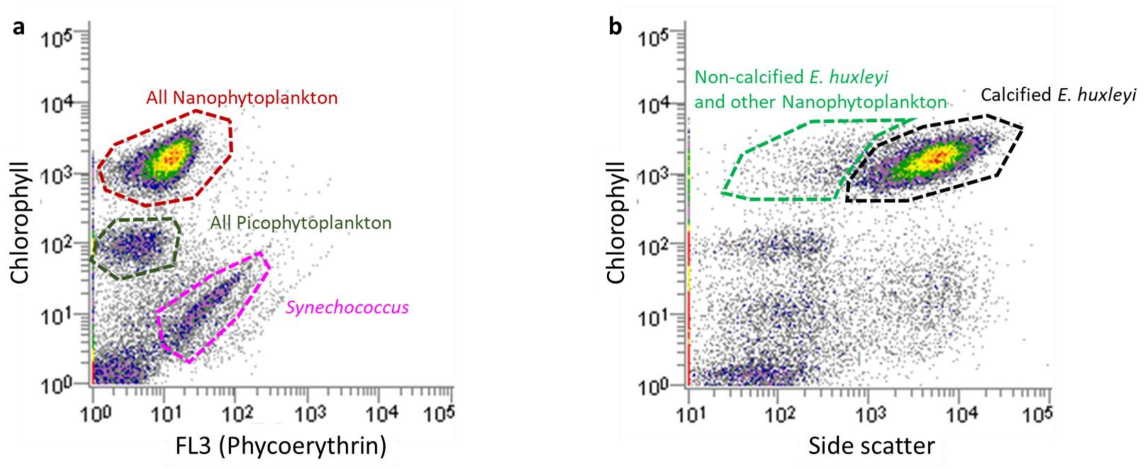

Figure S1. Gating strategy for flow cytometry analysis of natural phytoplankton communities. (a) Example cytogram that contains a mixture of Nanophytoplankton (red), Picophytoplankton (green) and Synechococcus (pink). Populations were identified based on their different chlorophyll and phycoerythrin content. (b) Calcified *E. huxleyi* was identified based on the high side scatter caused by the coccoliths that adorn the cells.
