# Supplementary material for: Ecological significance of extracellular vesicles in modulating host-virus interactions during algal blooms
Source: ISME J. 2021 Jun 4;15(12):3714–21. doi: 10.1038/s41396-021-01018-5 (PMC8630046; doi:10.1038/s41396-021-01018-5)
Supplement: Supplementary file 2 — Figure S2 [file 41396_2021_1018_MOESM2_ESM.pdf]

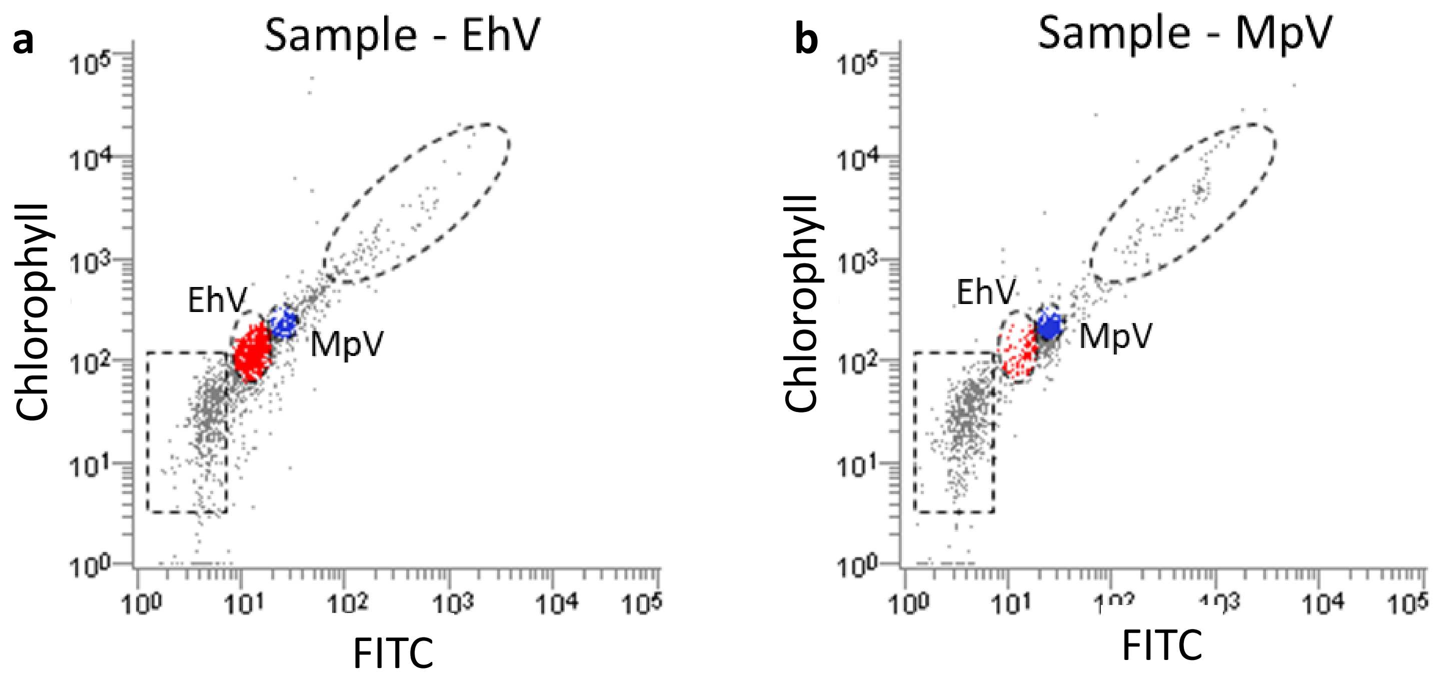

Figure S2. Gating strategy for counting EhV in mesocosm samples. The gates in the flow cytometer were set by using EhV and MpV samples from the lab. In a, a sample of EhV was analyzed and in b an MpV sample was analyzed. Particles that are counted towards EhV are in red and those counted as MpV are in blue. For both, the concentration of particles was similar to that of EhV during the demise phase of the natural bloom.
